# The Abnormal Functional Connectivity between the Hypothalamus and the Temporal Gyrus Underlying Depression in Alzheimer’s Disease Patients

**DOI:** 10.3389/fnagi.2018.00037

**Published:** 2018-02-13

**Authors:** Xiaozheng Liu, Wei Chen, Yunhai Tu, Hongtao Hou, Xiaoyan Huang, Xingli Chen, Zhongwei Guo, Guanghui Bai, Wei Chen

**Affiliations:** ^1^China-USA Neuroimaging Research Institute, Department of Radiology of the Second Affiliated Hospital and Yuying Children’s Hospital, Wenzhou Medical University, Wenzhou, China; ^2^Eye Hospital, Wenzhou Medical University, Wenzhou, China; ^3^Tongde Hospital of Zhejiang Province, Hangzhou, China; ^4^Department of Psychiatry, Sir Run Run Shaw Hospital, Zhejiang University School of Medicine and the Collaborative Innovation Center for Brain Science, Hangzhou, China; ^5^Key Laboratory of Medical Neurobiology of Chinese Ministry of Health, Hangzhou, China

**Keywords:** Alzheimer’s disease, depression, functional magnetic resonance imaging, functional connectivity, hypothalamus

## Abstract

Hypothalamic communication with the rest of the brain is critical for accomplishing a wide variety of physiological and psychological functions, including the maintenance of neuroendocrine circadian rhythms and the management of affective processes. Evidence has shown that major depressive disorder (MDD) patients exhibit increased functioning of the hypothalamic-pituitary-adrenal (HPA) axis. Neurofibrillary tangles are also found in the hypothalamus of Alzheimer’s disease (AD) patients, and AD patients exhibit abnormal changes in the HPA. However, little is known of how the hypothalamus interacts with other brain regions in AD patients with depression (D-AD). Functional connectivity (FC) analysis explores the connectivity between brain regions that share functional properties. Here, we used resting-state (rs) magnetic resonance imaging (MRI) technology and the FC method to measure hypothalamic connectivity across the whole brain in 22 D-AD patients and 21 non-depressed AD patients (nD-AD). Our results showed that D-AD patients had reduced FC among the hypothalamus, the right middle temporal gyrus (MTG) and the right superior temporal gyrus (STG) compared with the FC of nD-AD patients, suggesting that the abnormal FC between the hypothalamus and the temporal lobe may play a key role in the pathophysiology of depression in AD patients.

## Introduction

Symptoms of major depression of varying severity are a common comorbidity in Alzheimer’s disease (AD), with a prevalence of up to 63% in patients with AD (Khundakar and Thomas, 2015). In addition, these symptoms result in more rapid functional decline and loss of independence, and even shorter survival lengths (Lyketsos and Lee, 2004; Chi et al., 2015). Although both psychiatric and neurological disorders recognize the occurrence of affective and psychotic symptoms in patients with AD, the underlying mechanisms of depressive symptoms in these patients still remain unclear. Certain AD studies have emphasized psychosocial factors, such as functional and cognitive disabilities, whereas others have stressed neurobiological underpinnings. Improved knowledge of the neurobiological basis is required for the development of more effective treatment strategies (Khundakar and Thomas, 2015).

The current research considers the hypothalamo-pituitary-adrenal (HPA) axis, which mediates stress responses, one major pathway for depressive symptomatology (Schindler et al., 2012). HPA axis activity is governed by the secretion of adrenocorticotropic hormone-releasing factor (CRF) and vasopressin (AVP) from the hypothalamus, which, in turn, activates the secretion of adrenocorticotropic hormone (ACTH) from the pituitary, which ultimately stimulates the secretion of glucocorticoids from the adrenal cortex (Nemeroff, 1996). The neuropeptides, CRF and AVP, are released within the paraventricular nucleus (PVN) of the hypothalamus and are crucially involved in the pathogenesis of depression (Bao et al., 2008). Studies over the last 40 years have shown HPA axis hyperactivity as one of the most consistent biological findings in major depressive disorder (MDD). Meanwhile, with the development of medical imaging, numerous neuroimaging studies have investigated the neurobiological roles of the hypothalamus in MDD patients (Baeken et al., 2009; Gao et al., 2013; Sudheimer et al., 2015).

Baeken et al. (2009) examined the emotional and neurobiological effects of one session of high-frequency repetitive transcranial magnetic stimulation (HF-rTMS) applied to the left dorsolateral prefrontal cortex on a sample of unipolar treatment-resistant depressed patients of the melancholic subtype. To examine possible time delays in the HF-rTMS effects, mood and salivary cortisol were assessed not only immediately after the sessions but also after a period of 30 min. They found support for the hypothesis that a single session has a significant impact on the HPA axis, as measured by salivary cortisol. Additionally, Gao et al. (2013) found diminished GABAergic input to the hypothalamus upon postmortem examinations of MDD patients. Using resting-state functional magnetic resonance imaging (rsfMRI) and functional connectivity (FC) analyses, Sudheimer et al. (2015) reported that MDD patients show reduced FC between the hypothalamus and the subgenual cortex compared with the FC of healthy participants. Further, increased cortisol secretion and reduced connectivity were both found to be associated with MDD severity.

It is well known that hypothalamic communication with the rest of the brain is crucial for a wide variety of physiological and psychological functions, e.g., managing affective processes and maintaining neuroendocrine circadian rhythms. To accomplish these functions, the hypothalamus maintains neural connections within the brain and coordinates a variety of neuroendocrine cascades that influence target tissues throughout the body. However, little is known of how the hypothalamus interacts with other brain regions in mild AD patients with depression (D-AD). FC is defined as temporal correlations between spatially remote neurophysiological events or functional interactions (Büchel and Friston, 2000). Given that the hypothalamus may be significantly involved in MDD, we hypothesized that fMRI FC between the hypothalamus and emotional processing areas of the brain would be abnormal in D-AD patients compared with that of non-depressed AD (nD-AD) patients.

Thus, here we used the hypothalamus as a “seed” to investigate FC changes in D-AD patients and assessed the correlation between the FC changes and depressive symptom severity.

## Materials and Methods

### Patients

The sample group was composed of 21 nD-AD patients and 22 D-AD patients, recruited from Tongde Hospital in Zhejiang Province, China. Diagnoses were confirmed using the National Institute on Aging-Alzheimer’s Association guidelines (McKhann et al., 2011), with scores of 20–24 on the Mini-Mental State Examination (MMSE) and 1 on the Clinical Dementia Rating scale (CDR). Patients were screened to exclude those with histories of alcoholism, smoking, neurological disorders, or psychiatric disorders and those who were taking antidepressant medication. Patients were also excluded if the dual-echo MRI images showed two or more hyperintense lesions with diameters ≥5 mm or more than four hyperintense lesions with diameters 0–5 mm. All patients were right-handed, had more than 6 years of education and were 65–80 years old. This study was carried out in accordance with the recommendations of the Declaration of Helsinki and the principles of good clinical practice, the Ethics Committee of Tongde Hospital with written informed consent from all subjects. All subjects gave written informed consent in accordance with the Declaration of Helsinki. The protocol was approved by the the Ethics Committee of Tongde Hospital.

The diagnoses of depression were determined by two trained psychiatrists using the Diagnostic and Statistical Manual of Mental Disorders, fourth edition (DSM-IV; Gmitrowicz and Kucharska, 1994). In brief, all D-AD patients exhibited one or more of two core criteria (depressed mood, loss of interest, or pleasure) lasting for >2 weeks. Depression severity was evaluated using the Hamilton Depression Rating Scale (HAMD-17; Hamilton, 1967) and the Neuropsychiatric Inventory (NPI; Cummings et al., 1994). The scores on the HAMD-17 range between 7 and 17, and for the depression domain of the NPI (D-NPI), scores ≥4 are typically considered indicative of clinical significance (Schneider et al., 2001).

### MRI Scanning

MRI scanning was performed at mid day and patients were fasted for at least 6 h before MRI examination. Imaging data were acquired using a 3T Siemens scanner (Siemens Magnetom Verio; Siemens Medical Systems, Erlangen, Germany) at Tongde Hospital. All patients were placed in a birdcage head coil, with foam padding fitted to reduce head motion. rs fMRI scans were obtained using a gradient echo T2*-weighted sequence with the following parameters: 33 axial slices, thickness/gap = 4.8/0 mm, in-plane resolution = 64 × 64, repetition time (TR) = 2000 ms, echo time (TE) = 30 ms, flip angle = 90° and field of view (FOV) = 200 × 200 mm^2^. Each condition consisted of 200 functional volumes. During the functional runs, patients were instructed to remain awake with their eyes closed. Additionally, high-resolution T1-weighted whole brain magnetization prepared rapid gradient echo images were obtained using the following parameters: 128 sagittal slices, slice thickness/gap = 1/0 mm, in-plane resolution = 512 × 512, TR = 1900 ms, TE = 2.48 ms, inversion time (TI) = 900 ms, flip angle = 9° and FOV = 256 × 256 mm^2^.

### T1-weighted Images

To investigate the effects of gray matter (GM) volume on the FC analyses, we performed voxel-based morphometry on the T1-weighted images using the VBM8 toolbox in SPM8^1^. T1-weighted images were spatially normalized to the T1-weighted space (Montreal Neurological Institute, MNI^2^). Following this normalization, the resulting images were automatically segmented into GM, white matter (WM) and cerebrospinal fluid (CSF). Finally, the segmented images were nonlinearly modulated to compensate for spatial normalization effects, and individual GM volumes (GMVs) of the whole brain were calculated. The GMVs were compared between the two groups using two-sample two-tailed *t*-tests.

### rsfMRI Data Processing

All rsfMRI data preprocessing was performed using SPM8^1^ and the Data Processing Assistant for Resting State fMRI^3^ software. The preprocessing consisted of removing the first 10 volumes of the functional images, slice timing correction and motion correction. In regard to the motion correction, all participants had <1.5 mm maximum displacement in the x-, y-, or z-axes, with 1.5° of angular motion during the entire rsfMRI scan. Then, we compared the mean absolute displacement of head motion, and there was no significant difference between the two groups in regard to mean motion. The functional images were then coregistered to a high-resolution anatomical scan, normalized to the MNI space, and resampled at 3 mm^3^. The normalized images were smoothed using a Gaussian kernel of 6 mm^3^ full-width half-maximum (FWHM). Finally, temporal filtering was used to extract the signals in the 0.01–0.08-Hz frequency band, followed by linear regression to factor out six head motion parameters, along with the average CSF and WM signals.

### Seed-Based FC Analysis

In each individual rsfMRI data analysis, the hypothalamic region was defined according to a previous study (Baroncini et al., 2012). This provided a comprehensive atlas comparing anatomical, histological and MR images of the human hypothalamus and transferred each identified structure to the MNI space. The seed point was selected as (2, −1, −12) and was located in the PVN, which releases CRH and AVP. Seed spheres were constructed by drawing a 6-mm radius sphere around the seed point, with a time series for the seed sphere extracted from the preprocessed data. Seed-based rsFC analysis was performed using the temporal correlation approach. Time series were averaged across all voxels within each seed’s sphere. Pearson’s correlation analysis was performed between the seeds and the remaining voxels. The resulting values were transformed to Z values to improve their Gaussian distribution.

### Statistical Analysis

To explore the rsFC differences between the groups in the MNI standard space, second-level random effect two-sample *t*-tests, with the GMVs as covariates, were performed on the individual normalized FC maps in a voxel-by-voxel manner. AlphaSim, a program based on Monte Carlo simulations and implemented by Analysis of Functional NeuroImages (AFNI)^4^, was used to correct for multiple comparisons. Monte Carlo simulations determine the random distribution of the cluster size for a given per voxel threshold (Ledberg et al., 1998). According to this distribution, the statistical threshold was set at *P* < 0.05 and a cluster size >198 voxels, which corresponded to a corrected *P* < 0.05. The correction was confined within the GM mask and was determined by Monte Carlo simulations (Ledberg et al., 1998).

### Pearson’s Correlation Analysis of Hypothalamic FC

To determine whether the rsFC of the hypothalamus showed significant group differences that were correlated with the clinical variables, Pearson’s correlation analyses were performed between the Z-values from the abnormal brain regions and the clinical parameters of the D-AD and nD-AD patients in a voxelwise manner. The statistical threshold was set at *P* < 0.05 (after false discovery rate (FDR) correction; Ellis et al., 2000).

## Results

### Demographics and Clinical Characteristics

Overall, there were 22 AD patients in the D-AD group and 21 AD patients in the nD-AD group. The D-AD and nD-AD groups were well matched in terms of age (*t* = −1.414, *P* = 0.165), sex distribution (*χ*^2^ = 0.024, *P* = 1.000), and years of education (*t* = 0.757, *P* = 0.453). None of the patients were excluded according to our exclusion criteria. There was a significant difference in the HAMD-17 scores between the two groups (*t* = 14.253, *P* < 0.001). Details of the demographic data and corresponding tests are shown in Table 1.

**Table 1:** Demographic and neuropsychological data.

	D-AD	nD-AD	*t/χ*^2^	*p*
Gender, *n* (M/F)	22 (11/11)	21 (11/10)	0.024	1.000
Age, years	71.9 ± 4.5	73.9 ± 5.2	−1.414	0.165
Education, years	9.7 ± 2.2	9.1 ± 2.4	0.757	0.453
MMSE	20.9 ± 2.3	20.4 ± 1.7	0.787	0.436
HAMD	13.1 ± 2.4	3.8 ± 1.6	14.258	0.000
D-NPI	6.00 ± 1.6	0	–	–

*Data represent mean ± SD. Data were analyzed using independent sample *t*-tests. AD, Alzheimer’s disease; D-AD, Alzheimer’s disease patients with depressive symptoms; nD-AD, non-depressed AD patients; M, male; F, female; MMSE, Mini-Mental State Examination. D-NPI, depression domain of the Neuropsychiatric Inventory; HAMD, Hamilton Depression Rating Scale*.

### GM Volume

We found no whole brain GMV differences between the nD-AD patients and D-AD patients (two-tailed* t*-test, *t* = −0.5898, *P* = 0.5586).

### Abnormal Regional Brain Dysconnectivity Pattern in D-AD Patients

**Figure 1:**
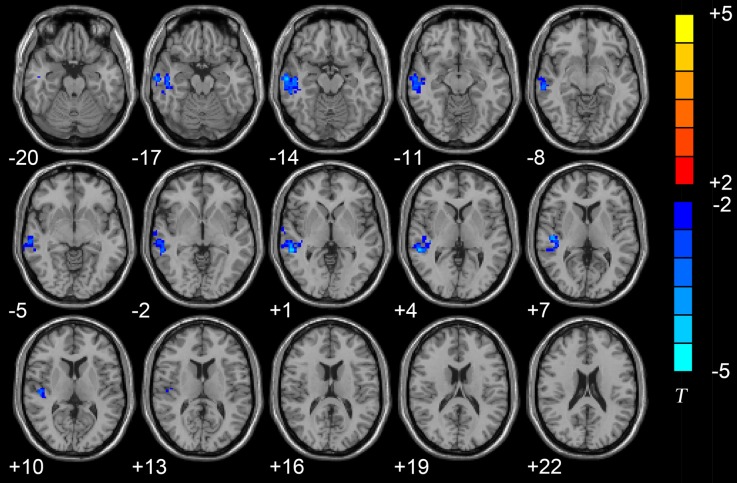
Axial brain region maps showing the decreased functional connectivity (FC) values in the depressed AD patients (D-AD) group compared with the non-depressed AD patients (nD-AD) group (*P* < 0.05, AlphaSim corrected). Results were viewed on the Montreal Neurological Institute (MNI) T1 template and the T-value scale is shown on the right of the image.

The results of the two-sample *t*-tests showed significant rsFC alterations in the related brain regions of the D-AD patients compared with the nD-AD patients (*P* < 0.05, AlphaSim corrected; Table 2). Specifically, we found that the D-AD patients exhibited decreased FC values, with peak differences in the right middle temporal lobe and the right superior temporal lobe (Figure 1, Table 2). All results were shown in the MNI template.

**Table 2:** Brain regions with significantly decreased functional connectivity (FC) values in the D-AD group compared with the nD-AD group.

Brain region	Voxels	BA	MNI coordinates			*T* value
			*x*	*y*	*z*
Right middle temporal lobe	293	21	57	−36	3	−3.1523
Right superior temporal lobe		22	50	−21	8	

*D-AD, AD patients with depressive symptoms; nD-AD, non-depressed AD patients; BA, Brodmann’s area; MNI, Montreal Neurological Institute*.

### Correlation between Hypothalamic FC and Neuropsychological Performance

No correlation was found between the MMSE, NPI, or HAMD scores and the hypothalamic FC in the nD-AD or the D-AD patients.

## Discussion

We used rsFC to examine the FC between the hypothalamus and the whole brain in AD patients. We found that, compared with the nD-AD patients, the D-AD patients exhibited reduced FC among the hypothalamus, the right middle temporal gyrus (MTG) and the right superior temporal gyrus (STG), suggesting that abnormal FC between the hypothalamus and the temporal lobe plays a key role in the pathophysiology of depression in AD patients. To the best of our knowledge, this is the first rsfMRI report showing disrupted hypothalamic FC in D-AD patients.

The evidence suggests that limbic-cortical-striato-pallido-thalamic structures organize emotional expression. Dysfunction within and between the structures in this circuit may induce disturbances in emotional behavior and in other cognitive aspects of the depressive syndrome in humans (Sheline, 2003; Drevets et al., 2008; Terroni et al., 2011). Lu et al. (2016) reported that first-episode, untreated MDD patients show significant volume reductions in the thalamus, while probabilistic tractography found that the deformed thalamic shape area had connections with the frontal and temporal lobes, which were related to major depression. Because limbic structures enable forebrain modulation of the hypothalamus and brainstem, their dysfunction can account for disturbances in autonomic regulation and neuroendocrine responses that are associated with mood disorders (Maletic et al., 2007). Several brain regions (including the temporal lobe) regulate emotional responses as circumstances and are known as top-down cognitive control mechanisms. Recent research suggests that reappraisal, a top-down emotion regulation strategy, is more effective in decreasing self-reported negative affect when emotions are generated in a top-down, vs. bottom-up manner (Otto et al., 2014; Morawetz et al., 2016). A few studies have shown GM abnormalities in the temporal cortex in treatment-resistant depression (TRD), treatment-responsive depression (TSD) and late-life depression (LLD) patients. Further, based on FC analysis using the right MTG as the seed, both the TRD and TSD patients show altered connectivity, mainly in the default-mode network (Ma et al., 2012; Harada et al., 2016). Studies of antidepressant treatment of LLD also showed that remission status is associated with right MTG changes (Khalaf et al., 2016; Karim et al., 2017).

Reduced FC between the hypothalamus and the temporal lobe has also been shown to promote the release of CRH due to abnormal inhibitory connections. The subsequent result is raised plasma adrenocorticotropic hormone levels, which in turn increase the number of GM lesions in the temporal lobe (Bennett, 2011). Based on animal experiments, Myers et al. (2016) confirmed that the hypothalamus represents an important stress-integration center, regulating behavioral processes and connecting the limbic forebrain to the neuroendocrine system. Moreover, the hypothalamus appears to be uniquely situated to play a role in stress-related pathologies associated with limbic-hypothalamic dysfunction. Dysfunction of the limbic-HPA (LHPA) system results from higher ACTH levels and contributes to disturbances in serotonergic and noradrenergic neurotransmission (Twardowska and Rybakowski, 1996). Coexistent dysregulation of the LHPA is predominantly linked to glucocorticoid receptor (GR) dysfunction within the limbic system. Along with hypercortisolemia, an imbalance of mineralocorticoid receptors (MR) and GR results in impaired negative feedback mechanisms in the LHPA loop. Impaired GR function and an MR/GR imbalance alters the negative feedback regulation within the LHPA, followed by the dysregulation and hypercortisolemia that is associated with decreased postsynaptic 5-HT1A receptor activity, thereby resulting in serotoninergic dysfunction (Lesch et al., 1990).

The MTG is located in the extended dorsal attention system and is involved in cued attention and working memory (Corbetta and Shulman, 2002; Fox et al., 2006). Using an optimized voxel-based method, Peng et al. (2011) reported reduced GMV in the bilateral MTG in a group of first-episode MDD patients. In addition, Wu et al. (2011) reported that TRD patients show higher regional homogeneity in the right MTG than those of treatment non-resistant depression patients and healthy controls. Moreover, lower amplitude low-frequency fluctuation values in this region were found to be reduced in both TRD and TSD patients (Guo et al., 2012). These findings suggest that the MTG is part of a relevant functional network associated with MDD.

The STG consists of the primary auditory cortex and the auditory association areas (Pearlson, 1997; Kim et al., 2000; Hou et al., 2016) and has been implicated in emotional processing and social cognition (Allison et al., 2000; Arnsten and Rubia, 2012). A recent meta-analysis of fMRI studies of MDD noted that the STG is one of the most consistently identified regions involved in its pathophysiology (Fitzgerald et al., 2008). Takahashi et al. (2010) delineated STG subregions (namely, the planum polare, planum temporale, rostral STG, caudal STG and Heschl’s gyrus) and the temporal pole using MRI in 29 currently depressed patients, 27 remitted depressed patients, and 33 age- and gender-matched healthy control subjects. Both the current and remitted MDD patients showed significant volume reductions in the left planum temporale and the bilateral caudal STG compared with healthy controls. Guo et al. (2011) used a regional homogeneity approach to explore the brain activity features of TRD patients. Compared with healthy controls, decreased regional homogeneity was found in the TRD patients in the left insula, STG, inferior frontal gyrus, lingual gyrus and anterior cerebellar lobe. Our finding showing reduced FC between the hypothalamus and the STG in D-AD patients is consistent with those of previous studies, suggesting that abnormal STG activity may be associated with negative emotional processing.

Several limitations should be considered when interpreting our results. First, the hypothalamus encompasses a relatively small volume; thus, the accuracy of the seed point location depends on the spatial resolution of the fMRI images and the accuracy of the registration method. Higher spatial resolution fMRI images and a higher accuracy registration method should be used to improve the accuracy of the seed point location (Klein et al., 2009; Yacoub et al., 2015). Second, a group of MDD patients should be included in future studies. Comparisons between MDD and D-AD patients can provide more information regarding the pathophysiology of depression in AD patients. Third, in this study, in consideration of the dysregulation of the HPA-axis involved in both MDD and AD (Sudheimer et al., 2015) and the critical role of the PVN in the dysregulation of the HPA-axis, we choose the PVN as the seed point for the FC analyses of the whole brain. Meanwhile, both the AVP and CRH released by the HPA-axis contribute to the signs and symptoms of depression; thus, detecting the hormone concentrations of AVP, CRH and ACTH will be one of our future studies. Third, we used a relatively small sample; therefore, our statistical power was low and limited. Future studies should use a larger sample size to increase the statistical power. Finally, there are issues of the sample, where they were not matched on body weight or Body Mass Index, which may be one of the factors that can affect the brain function network in AD patients (Sugimoto et al., 2017).

## Conclusion

Here, we used rsfMRI and rsFC analysis to examine the intrinsic dysconnectivity patterns of the hypothalamus in both D-AD and nD-AD patients. We found decreased FC in the right middle temporal lobe and the right superior temporal lobe. These findings enhanced our understanding of hypothalamic dysfunction in D-AD patients.

## Author Contributions

XL was responsible for data acquisition and analysis, and drafting of the manuscript. ZG, XH and WC were responsible for design/conception of the study, and data acquisition and analysis. GB and YT was responsible for drafting the manuscript and critical revision of the manuscript for intellectual content. HH was responsible for data acquisition and analysis. WC and XC were responsible for analysis and interpretation of the data. All authors agree to be accountable for all aspects of the work.

## Conflict of Interest Statement

The authors declare that the research was conducted in the absence of any commercial or financial relationships that could be construed as a potential conflict of interest.

## Footnotes

^1^http://www.fil.ion.ucl.ac.uk/spm

^2^http://www.bic.mni.mcgill.ca

^3^http://www.restfmri.net

^4^http://afni.nimh.nih.gov
